# Expression of p16^INK^ ^4a^ is a biomarker of chondrocyte aging but does not cause osteoarthritis

**DOI:** 10.1111/acel.12771

**Published:** 2018-05-09

**Authors:** Brian O. Diekman, Garrett A. Sessions, John A. Collins, Anne K. Knecht, Susan L. Strum, Natalia K. Mitin, Cathy S. Carlson, Richard F. Loeser, Norman E. Sharpless

**Affiliations:** ^1^ Lineberger Comprehensive Cancer Center University of North Carolina School of Medicine Chapel Hill North Carolina; ^2^ Thurston Arthritis Research Center University of North Carolina School of Medicine Chapel Hill North Carolina; ^3^ Department of Biomedical Engineering University of North Carolina, Chapel Hill, NC North Carolina State University Raleigh North Carolina; ^4^ HealthSpan Diagnostics LLC Research Triangle Park North Carolina; ^5^ Department of Veterinary Clinical Sciences University of Minnesota St. Paul Minnesota; ^6^ Division of Rheumatology, Allergy, and Immunology University of North Carolina School of Medicine Chapel Hill North Carolina; ^7^ Departments of Medicine and Genetics University of North Carolina School of Medicine Chapel Hill North Carolina; ^8^ The National Cancer Institute Bethesda Maryland

**Keywords:** aging, cellular senescence, chondrocyte, geroscience, Ink4a, mouse models, osteoarthritis, p16

## Abstract

Cellular senescence drives a functional decline of numerous tissues with aging by limiting regenerative proliferation and/or by producing pro‐inflammatory molecules known as the senescence‐associated secretory phenotype (SASP). The senescence biomarker *p16* ^*INK*^ ^*4a*^ is a potent inhibitor of the cell cycle but is not essential for SASP production. Thus, it is unclear whether *p16* ^*INK*^ ^*4a*^ identifies senescence in hyporeplicative cells such as articular chondrocytes and whether *p16* ^*INK*^ ^*4a*^ contributes to pathologic characteristics of cartilage aging. To address these questions, we examined the role of *p16* ^*INK*^ ^*4a*^ in murine and human models of chondrocyte aging. We observed that *p16* ^*INK*^ ^*4a*^ mRNA expression was significantly upregulated with chronological aging in murine cartilage (~50‐fold from 4 to 18 months of age) and in primary human chondrocytes from 57 cadaveric donors (*r* ^2 ^= .27, *p* < .0001). Human chondrocytes exhibited substantial replicative potential in vitro that depended on the activity of cyclin‐dependent kinases 4 or 6 (CDK4/6), and proliferation was reduced in cells from older donors with increased *p16* ^*INK*^ ^*4a*^ expression. Moreover, increased chondrocyte *p16* ^*INK*^ ^*4a*^ expression correlated with several SASP transcripts. Despite the relationship between *p16* ^*INK*^ ^*4a*^ expression and these features of senescence, somatic inactivation of *p16* ^*INK*^ ^*4a*^ in chondrocytes of adult mice did not mitigate SASP expression and did not alter the rate of osteoarthritis (OA) with physiological aging or after destabilization of the medial meniscus. These results establish that *p16* ^*INK*^ ^*4a*^ expression is a biomarker of dysfunctional chondrocytes, but that the effects of chondrocyte senescence on OA are more likely driven by production of SASP molecules than by loss of chondrocyte replicative function.

## 1 INTRODUCTION

Cellular senescence plays an instrumental role in limiting the development of pathological states including cancer, but the accumulation of senescent cells with aging also contributes to age‐related tissue dysfunction (He & Sharpless, 2017). Cellular senescence is a complex phenotype characterized by two arms: a stress‐induced, durable cell cycle arrest and the production of a suite of pro‐inflammatory molecules known as the senescence‐associated secretory phenotype (SASP) (Childs, Durik, Baker & van Deursen, 2015; Coppe, Desprez, Krtolica & Campisi, 2010; Munoz‐Espin & Serrano, 2014). The senescence biomarker p16^INK4a^ mediates cell cycle arrest through inhibition of cyclin‐dependent kinase 4 and 6 (CDK4/6), but *p16* ^*INK4a*^ expression is not required for production of the SASP (Coppe et al., 2011). Furthermore, in vivo evidence suggests that the primary functional consequence of high *p16* ^*INK4a*^ expression with aging is to limit the proliferation of specific cell types during homeostasis or in response to injury (Janzen et al., 2006; Krishnamurthy et al., 2006; Liu et al., 2011; Molofsky et al., 2006; Sousa‐Victor et al., 2014). Several groups, however, have suggested cell cycle independent effects of p16^INK4a^ and CDK4/6 inhibition (Goel et al., 2017; Murakami, Mizoguchi, Saito, Miyasaka & Kohsaka, 2012), and it is unclear whether reduced *p16* ^*INK4a*^ expression can protect tissues from age‐related pathologies that are associated with the SASP but not with replicative failure.

Chondrocytes are metabolically active and synthesize cartilage matrix throughout adulthood. These cells exhibit an interesting replicative biology, showing extremely low proliferation rates in adult humans and mice during homeostasis (Aigner et al., 2001; Decker et al., 2017). Chondrocyte proliferation can occur during the development of osteoarthritis (OA) in the form of cell clusters, but it is not known if this replicative response is regenerative, pathologic, or epiphenomenal (Lotz et al., 2010). Chondrocytes also display features of senescence with aging and OA, likely in response to macromolecular damage that accumulates in these long‐lived cells (Martin & Buckwalter, 2001; McCulloch, Litherland & Rai, 2017; Price et al., 2002; Rose et al., 2012). OA progression is driven by inflammatory cytokines that initiate a cascade of matrix degradation, and the prominent role for SASP factors such as matrix metalloproteinase 13 (MMP‐13) and interleukin 1 alpha (IL‐1α) implicates senescent cells in the joint as a source of these catabolic molecules (Loeser, Collins & Diekman, 2016). The functional role of p16^INK4a^ in cartilage aging and OA is less clear, although knockdown and overexpression studies in cultured chondrocytes have confirmed that *p16* ^*INK4a*^ expression is associated with the production of catabolic factors involved in dedifferentiation and OA (Philipot et al., 2014; Zhou, Lou & Zhang, 2004). Further evidence that non‐cell‐autonomous effects of senescence contribute to OA has come from a recent study showing that selective elimination of senescent cells can mitigate the development of OA (Jeon et al., 2017). This “senolytic” approach seeks to target senescent cells that are marked by high expression of *p16* ^*INK4a*^, in line with findings from related murine models showing that the depletion of *p16* ^*INK4a*^‐high cells in old animals can ameliorate features of aging and extend lifespan (Baker et al., 2011, 2016; Kirkland, Tchkonia, Zhu, Niedernhofer & Robbins, 2017).

The largely hyporeplicative nature of chondrocytes as well as the association between senescence and OA encouraged us to pursue murine and human studies to directly address the role of *p16* ^*INK4a*^ expression in cartilage aging. Toward that end, we studied *p16* ^*INK4a*^ expression and proliferation in cultured chondrocytes and employed a Cre recombinase driven approach to examine the effects of somatic *p16* ^*INK4a*^ inactivation in well‐defined murine models of physiological and injury‐driven OA.

## 2 RESULTS

### 2.1 Increased p16^INK4a^ gene expression in murine and human articular cartilage with aging

**Figure 1:**
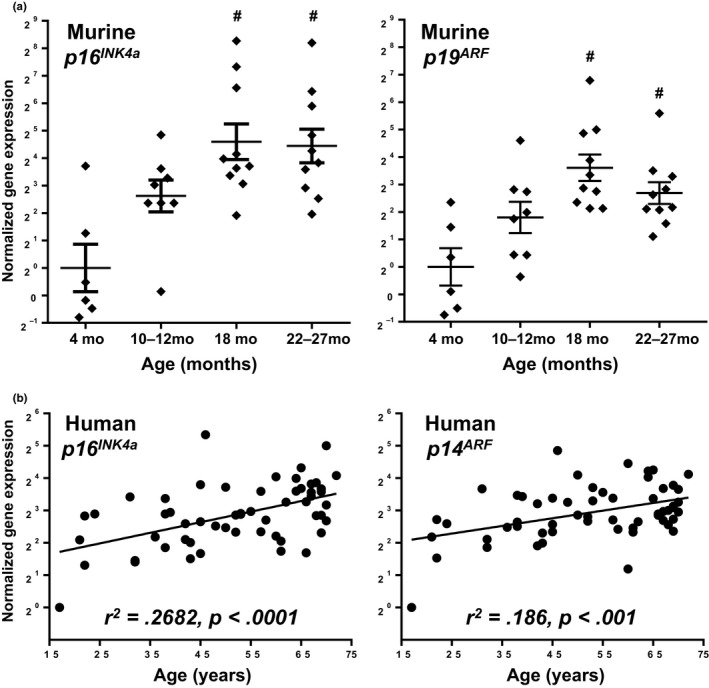
Gene expression in murine and human cartilage with age. (a) *p16* ^*INK*^ ^*4a*^ (left) and *p19* ^*ARF*^ (right) gene expression of hip cartilage from wild‐type mice. *n* ≥ 6 per group; mean ± *SEM*; ^#^ *p* < .05 to 4 months by ANOVA, Tukey's post hoc. (b) Gene expression in primary human chondrocytes from 57 cadaveric donors. *R* ^2^ and *p* values from linear regression analysis shown

We determined the expression of *p16* ^*INK4a*^ with aging in murine chondrocytes by performing quantitative RT–PCR on cartilage tissue isolated from the proximal femur. In accord with observations in other murine tissues (Krishnamurthy et al., 2004), there was a significant increase in *p16* ^*INK4a*^ expression (48.4‐ and 43.5‐fold, respectively) in the 18‐month and 22‐ to 27‐month groups as compared to skeletally mature mice of 4 months of age (*p* < .01, Figure 1a). A significant but less robust upregulation was seen in the related gene product *p19* ^*ARF*^, with an average fold increase of 24.4‐ and 12.9‐fold at 18 months and 22–27 months of age, respectively (*p* < .01, Figure 1a). These data support the concept that the physiological stresses of aging induce expression of the *Ink4a/Arf* locus, even in a hyporeplicative cell type such as chondrocytes. To determine whether aged murine chondrocytes display other characteristic features of senescent cells, we assessed senescence‐associated β‐galactosidase (SA β‐gal) activity (Dimri et al., 1995). Consistent with reports in human chondrocytes (Martin & Buckwalter, 2001), chondrocytes from 22‐ to 24‐month‐old mice displayed a higher percentage of SA β‐gal‐positive cells as compared to 5‐ to 9‐month‐old mice (*p* = .03, Figure S1).

The expression of *p16* ^*INK4a*^ with aging was also evaluated in primary human chondrocytes isolated from the cartilage of 57 cadaveric donors without evidence of OA. The donors were between 17 and 72 years old and age was responsible for 27% of the variation in *p16* ^*INK4a*^ levels (Figure 1b, *r* ^2 ^= .2682, *p* < .0001). The expression of *ARF* (denoted as *p14* ^*ARF*^ for human cells) was also significantly correlated with age (Figure 1b, *r* ^2 ^= .186, *p* < .001) but to a lesser extent than *p16* ^*INK4a*^. Expression of *p16* ^*INK4a*^ and *p14* ^*ARF*^ showed a strong correlation with each other (*r* ^2 ^= .6962, *p* < .001, data not shown). The expression of additional candidate genes with potential involvement in senescence or cell cycle inhibition was examined for a relationship to age, but only *p16* ^*INK4a*^ and *p14* ^*ARF*^ showed a significant correlation to age (Table 1). Thus, the analysis of human chondrocytes suggests that *INK4a/ARF* expression is particularly affected by the process of chondrocyte aging.

**Table 1:** Expression of candidate aging biomarkers in human articular chondrocytes. An initial cohort of 37 cadaveric donors ranging in age from 21 to 70 years old were evaluated for gene expression. Expression was normalized to YWHAZ (custom assay) as a housekeeping gene and analyzed in log_2_ format. Linear regression analysis to age was performed with *r* ^2^ and *p* values shown. Significant correlations are indicated by as asterisk (*) and ampersand (&) notes that 11 samples with *C*_t_ values higher than 37 were set to 37 for analysis. CDKN, Cyclin‐dependent kinase inhibitor

Gene name (symbol)	Taqman Assay ID	*r* ^2^/*P* value to age
*p16* ^*INK4a*^ (*CDKN2A* variant 1)	Custom assay	.2058/<.01*
*p14* ^*ARF*^ (*CDKN2A* variant 4)	Custom assay	.1283/<.05*
p15 (*CDKN2B*)	Hs00793225_m1	.0403/>.05
p21 (*CDKN1A*)	Hs00355782_m1	.0982/>.05
p27 (*CDKN1B*)	Hs01597588_m1	.0032/>.05
Cyclin D1 (*CCND1*)	Hs00765553_m1	.0150/>.05
Interleukin 1β (*IL‐1*β)	Hs00174097_m1	.0159/>.05^&^
Interleukin 6 (*IL‐6*)	Hs00985639_m1	.0007/>.05
Interleukin 8 (*IL‐8*)	Hs00174103_m1	.0086/>.05
Matrix metalloproteinase 1 (*MMP‐1*)	Hs00899658_m1	.0465/>.05
Matrix metalloproteinase 3 (*MMP‐3*)	Hs00968305_m1	.0005/>.05
Matrix metalloproteinase 13 (*MMP‐13*)	Hs00942584_m1	.0013/>.05
Insulin‐like growth factor binding protein (*IGFBP3*)	Hs00426289_m1	.0037/>.05
Plasminogen activator inhibitor 1 (*PAI‐1/SERPINE1*)	Hs01126606_m1	.0189/>.05
Monocyte chemoattractant protein 1 (*MCP‐1/CCL2*)	Hs00234140_m1	.0259/>.05
Vascular endothelial growth factor A (*VEGFA*)	Hs00900055_m1	.002/>.05
Aggrecan (*ACAN*)	Hs00153936_m1	.0044/>.05
Interleukin 1α (*IL‐1*α)	Hs00174092_m1	Undetectable (expression in only six samples)
Interferon gamma (*IFNg*)	Hs00989291_m1	Undetectable (no expression in any samples)

### 2.2 In vitro human chondrocyte proliferation is controlled by multiple cyclin‐dependent kinases

**Figure 2:**
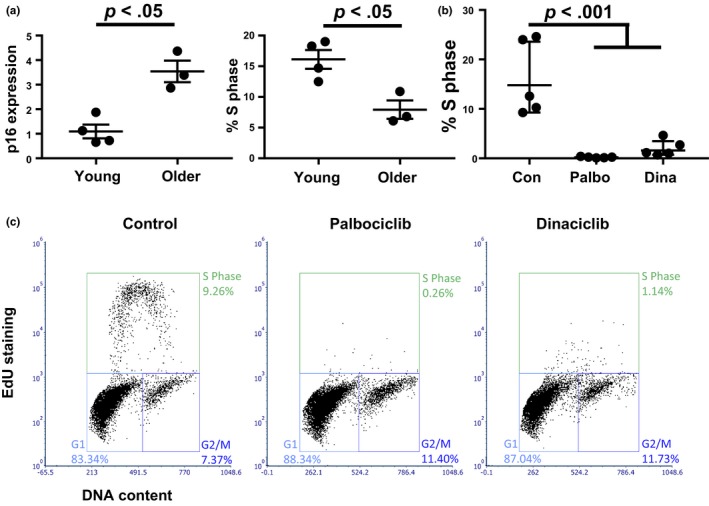
Effect of cyclin‐dependent kinase (CDK) inhibition on human chondrocyte proliferation. (a) A set of young and older (24.25 ± 2.6 vs. 64 ± 2.1 years old) donors were analyzed for *p16* ^*INK*^ ^*4a*^ gene expression (left) and the percentage of cells in S phase during monolayer culture (right). *p* Value shown by *t* test. (b) S phase percentage in chondrocytes from five donors treated with vehicle control, 1 μm Palbociclib, or 50 nm Dinaciclib. *p* Value shown by ANOVA with Tukey's post hoc. (c) Representative flow cytometry plots for percentage S phase calculation after a 4‐hr pulse of 5‐ethynyl‐2′‐deoxyuridine (EdU)

The proliferation of human chondrocytes is restrained in vivo by the dense extracellular matrix of cartilage, but these cells retain the potential for proliferation upon culture in low density monolayer conditions. To determine whether increased *p16* ^*INK4a*^ expression with aging has the potential to alter chondrocyte proliferation, we analyzed cell cycle entry in a set of young and older chondrocyte donors (24.25 ± 2.6 vs. 64 ± 2.1 years old) using a pulse of 5‐ethynyl‐2′‐deoxyuridine (EdU). In this cohort, older donors had a 3.5‐fold average increase in *p16* ^*INK4a*^ gene expression (Figure 2a, left, *p* < .05) and displayed a significantly reduced number of cells in S phase as compared to the younger donors (Figure 2a, right, 7.9% vs. 16.1%, *p* < .05). To explore a potential mechanistic role for p16^INK4a^ in regulating proliferation in this setting, we used pharmacological inhibition of CDK4/6 to mimic high expression of *p16* ^*INK4a*^. Palbociclib inhibits the binding of CDK4/6 to D type cyclins and has been developed as a promising therapeutic for cancers with altered function of the Cyclin D‐CDK4/6‐p16^INK4a^ pathway (Fry et al., 2004; Otto & Sicinski, 2017). Palbociclib completely inhibited the proliferation of human chondrocytes (*p* < .001, Figure 2b, c). Inhibition of CDK1/2/5/9 with Dinaciclib was similar to Palbociclib in preventing cell cycle entry, indicating that human chondrocytes are sensitive to inhibition of multiple CDKs. These results suggest that aging reduces the potential for chondrocytes to enter S phase when stimulated in monolayer culture, and that these effects on proliferation could be caused by altered regulation of several pathways including the Cyclin D‐CDK4/6‐p16^INK4a^ axis.

### 2.3 p16^INK4a^ expression correlates with SASP factors independent of chronological aging

**Figure 3:**
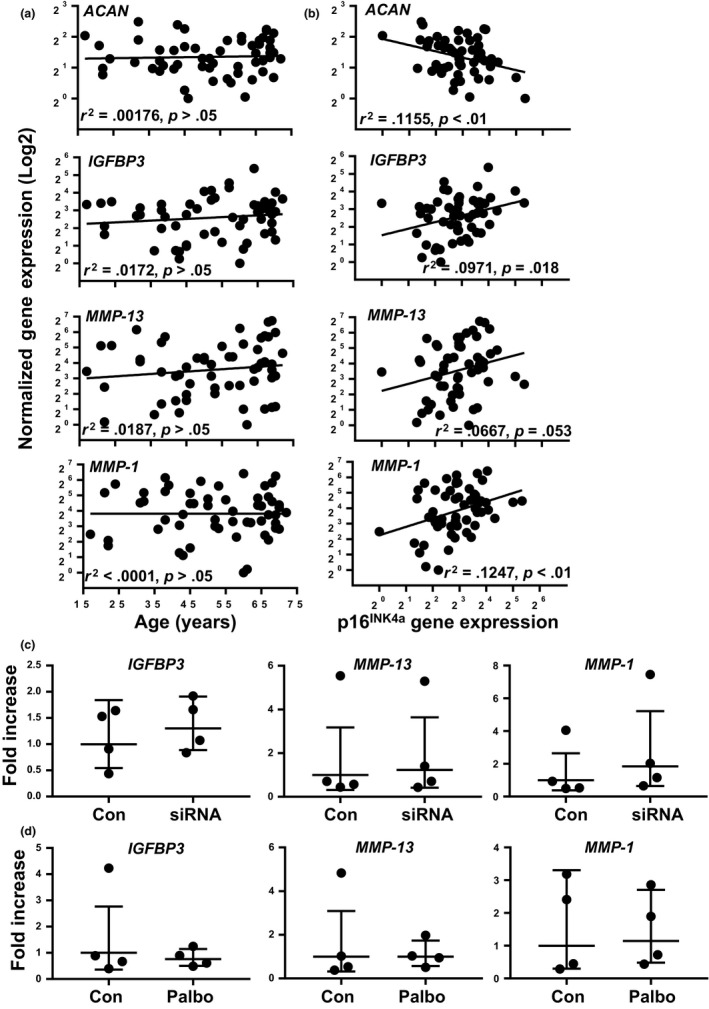
Gene expression in primary human chondrocytes. Data from 57 donors aged 17–72 years old are presented as a function of (a) age or (b) *p16* ^*INK*^ ^*4a*^ gene expression. The lowest value for each plot was set to 1. *R* ^2^ and *p* values from linear regression analysis shown. ACAN, Aggrecan; IGFBP3, insulin‐like growth factor binding protein 3; MMP, matrix metalloproteinase. (c) The effect of scrambled control or siRNA targeting *p16* ^*INK*^ ^*4a*^ and *p14* ^*ARF*^ on gene expression. (d) The effect of Palbociclib treatment on gene expression. In panels (c) and (d), data were normalized to control and all comparisons by paired *t* test were not significant (*p* > .05)

In addition to increased expression with chronological age, *p16* ^*INK4a*^ responds to physiological demands that accelerate the rate of aging. This has allowed *p16* ^*INK4a*^ expression to serve as a biomarker of molecular aging, which can be used to measure the senescence burden and predict cellular function in some settings (Koppelstaetter et al., 2008; Liu et al., 2009; Wood et al., 2016). To determine this relationship in primary human chondrocytes, we analyzed whether the expression of *p16* ^*INK4a*^ was associated with markers of chondrocyte dysfunction independent of chronological aging (Figure 3). Expression of several SASP markers showed a positive correlation to *p16* ^*INK4a*^ levels despite no correlation to age: insulin‐like growth factor binding protein 3 (*IGFBP3*,* r* ^2 ^= .0971, *p* = .018), matrix metalloproteinase 1 (*MMP‐1*,* r* ^2 ^= .1247, *p* < .01), and a strong trend for *MMP‐13* (*r* ^2 ^= .0667, *p* = .054). The expression of Aggrecan (*ACAN*), which is a positive marker of extracellular matrix synthesis, showed a significant reduction in donors with high levels of *p16* ^*INK4a*^ expression (*r* ^2 ^= .1155, *p* < .01). This gene expression pattern suggests that *p16* ^*INK4a*^ levels may represent the degree of dysfunction in human chondrocytes.

We analyzed the expression of SASP genes that showed a positive correlation to *p16* ^*INK4a*^ levels in the context of *INK/ARF* knockdown. Within one day of transfection, pooled siRNA targeting both genes reduced expression of *p16* ^*INK4a*^ and ARF to undetectable levels and less than 10% expression of scrambled siRNA controls, respectively. The expression of *IGFBP‐3*,* MMP‐1*, and *MMP‐13* was unaffected by knockdown of *p16* ^*INK4a*^ and *ARF* (Figure 3c). When Palbociclib was used to mimic *p16* ^*INK4a*^‐mediated cell cycle arrest, these same SASP factors were also unaffected (Figure 3d). These data support the concept that while SASP markers correlate with *p16* ^*INK4a*^ expression, *p16* ^*INK4a*^ or cell cycle arrest is not required for production of the SASP.

### 2.4 Somatic inactivation of p16^INK4a^ in murine chondrocytes

Given the correlation of *p16* ^*INK4a*^ expression with chronological aging and the SASP, as well as the potential for CDK4/6 inhibition to restrain chondrocyte proliferation, we sought to determine the effects of somatic *p16* ^*INK4a*^ loss in the chondrocyte compartment. Toward that end, we utilized Cre recombinase to eliminate exon 1α of *p16* ^*INK4a*^ in chondrocytes in vivo at skeletal maturity using a previously reported floxed *p16* ^*INK4a*^ allele (p16^L^; Monahan et al., 2010) and an inducible, chondrocyte‐specific Aggrecan Cre driver (*Acan* ^*tm1(cre/ERT2)Crm*^; Henry et al., 2009). A loxP‐stop‐loxP ZsGreen fluorescent reporter allele was also included in a subset of mice to facilitate fluorescent activated cell sorting (FACS) of chondrocytes that had undergone Cre‐mediated recombination. To test the efficacy of this system, chondrocytes were isolated by FACS 1 month after tamoxifen injection, showing undetectable levels of *p16* ^*INK4a*^ by qPCR in cells sorted from *Acan* ^*tm1(cre/ERT2)Crm*^ *p16* ^*L/L*^ (p16^INK4a^ loss) mice, with retained expression in *Acan* ^*tm1(cre/ERT2)Crm*^ *p16* ^*INK4a+/+*^ (p16^INK4a^ intact) controls (Figure S2A). The consistency of recombination in the articular cartilage after tamoxifen injection was demonstrated with anti‐tdTomato immunohistochemistry in mice containing *Acan* ^*tm1(cre/ERT2)Crm*^ and loxP‐stop‐loxP tdTomato fluorescent reporter alleles (Figure S2B).

We examined the effects of *p16* ^*INK4a*^ loss on the expression of chondrocyte mRNA markers, expecting little or no effect of *p16* ^*INK4a*^ deletion in young mice due to the low expression at this age. To determine whether *p16* ^*INK4a*^ loss would affect any age‐related increase in SASP factor production, we also analyzed gene expression of murine chondrocytes from 9‐ to 18‐month‐old animals. *Mmp‐13* showed significantly increased expression in older mice (*p* < .001, Figure S2C), but there was no effect of *p16* ^*INK4a*^ loss in either age group. Expression of *Igfbp3* showed a similar trend but was not statistically significant. In addition to gene expression, Mmp‐13 protein was detected by immunohistochemistry in joints with and without *p16* ^*INK4a*^ loss at 18 months (Figure S2D). These data indicate that *p16* ^*INK4a*^ can be efficiently deleted from CreER^T2^‐expressing chondrocytes via tamoxifen induction in adult mice, but that this deletion does not inhibit the increased production SASP factors during physiologic aging in mice.

The somatic loss of *p16* ^*INK4a*^ can initiate increased cell division and cancer in a cell‐type‐specific fashion (Liu et al., 2011), leading us to investigate chondrocyte proliferation and neoplasia in this cohort. We did not observe neoplasia in the articular cartilage, but did note a high rate of medullary neoplasia accompanied by abundant intramedullary bone formation in *Acan* ^*tm1(cre/ERT2)Crm*^ *p16* ^*L/L*^ mice (Figure S3). This neoplastic process did not appear to alter the histological features of the articular cartilage or underlying subchondral bone, but may have had indirect effects on joint function. To determine whether *Acan* ^*tm1(cre/ERT2)Crm*^‐driven loss of *p16* ^*INK4a*^ initiated widespread proliferation of articular chondrocytes at the joint surface, we provided BrdU through the drinking water for long‐term pulsing experiments. Articular chondrocytes with *p16* ^*INK4a*^ loss remained refractory to proliferation even in the context of cartilage damage induced by destabilization of the medial meniscus (DMM) that might stimulate attempted repair, as 3 weeks of continuous BrdU treatment only marked areas of early osteophyte formation (Figure S4A). This low rate of proliferation is one reason that *Acan* ^*tm1(cre/ERT2)Crm*^‐driven recombination persists in the articular cartilage over time, as demonstrated by immunohistochemistry targeting the product of a loxP‐stop‐loxP reporter allele 6 months after initiating recombination with tamoxifen (Figure S4B). These data suggest that articular chondrocytes exhibit little if any proliferation in adult murine cartilage, even in the setting of *p16* ^*INK4a*^ loss.

We also used in vitro culture of murine chondrocytes to explore the effect of *p16* ^*INK4a*^ loss on expansion rate and the development of replicative senescence. In these studies, we isolated chondrocytes from 3‐week‐old mice and expanded the cells through four passages. Cells with *p16* ^*INK4a*^ loss showed similar expansion rates and lost replicative potential at the same passage as chondrocytes from littermate controls that retained *p16* ^*INK4a*^ expression (Figure S5A). Additional features of senescence such as extensive staining for SA β‐gal (Figure S5B) and increased expression of the SASP marker *Igfbp3* were also not affected by somatic loss of *p16* ^*INK4a*^ (Figure S5C).

### 2.5 Somatic loss of p16^INK4a^ in chondrocytes does not protect mice from OA

**Figure 4:**
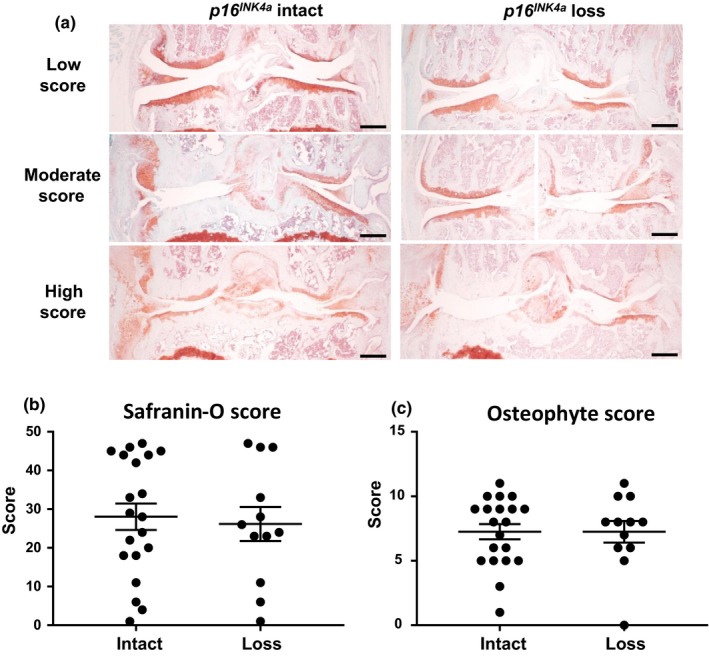
Effect of *p16* ^*INK*^ ^*4a*^ loss on spontaneous age‐related OA. (a) Histological sections of hindlimbs from 18‐month‐old mice were stained with Safranin‐O (red, glycosaminoglycans) and Fast Green (green, collagen). Representative images of mice with mild, moderate, and high total joint Safranin‐O scores from both *p16* ^*INK*^ ^*4a*^ intact and *p16* ^*INK*^ ^*4a*^ loss groups are shown. Scale bars = 200 μm. Sections were scored by a blinded observer for (b) the degree of Safranin‐O staining loss (high = OA, max score = 48) and (c) the size of osteophytes (high = OA, max score = 12). Analysis by Mann–Whitney test showed no significant difference between groups (*p* > .05) for either measure

The functional effects of *p16* ^*INK4a*^ loss in chondrocytes in vivo were further explored by analyzing the extent of age‐related OA in animals with or without *p16* ^*INK4a*^ deletion in chondrocytes. Littermate cohorts of *Acan* ^*tm1(cre/ERT2)Crm*^ *p16* ^*INK4a+/+*^ and *Acan* ^*tm1(cre/ERT2)Crm*^ *p16* ^*L/L*^ male mice were treated with tamoxifen at 4 and 12 months of age to induce *p16* ^*INK4a*^ loss. The development of OA was evaluated in mice sacrificed at 18 months of age using established histological scoring systems based on the loss of Safranin‐O staining and osteophyte formation in the load‐bearing cartilage of the femoral condyle and tibial plateau (McNulty et al., 2011). Spontaneous age‐related OA occurs with variable progression in male C57BL/6 mice, and our histological results underscored this variability by demonstrating mild, moderate, and severe OA at 18 months of age (Figure 4a). The Safranin‐O staining scores showed a similar distribution in both cohorts, indicating that inducing *p16* ^*INK4a*^ loss in chondrocytes is insufficient to prevent cartilage degradation with age (*p* > .05, Figure 4b). The effect on osteophyte formation, which is another marker of OA development in both human and murine joints, also showed no change with somatic inactivation of *p16* ^*INK4a*^ (*p* > .05, Figure 4c).

**Figure 5:**
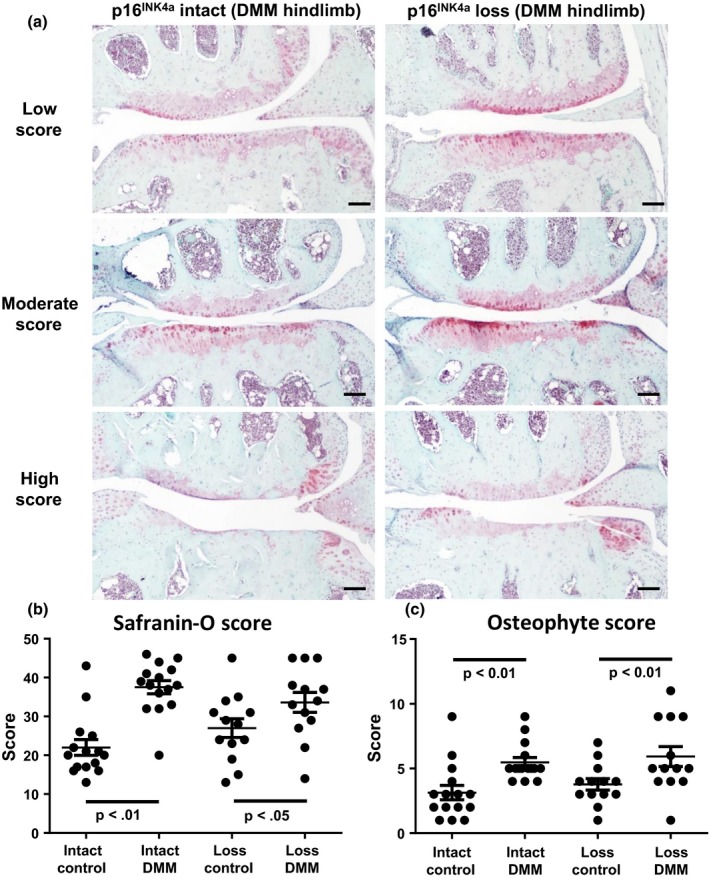
Effect of *p16* ^*INK*^ ^*4a*^ loss on injury‐induced OA. (a) Histological sections of hindlimbs from mice 8 weeks after destabilization of the medial meniscus (DMM) surgery were stained with Safranin‐O (red, glycosaminoglycans) and Fast Green (green, collagen). Representative images of the medial side of DMM hindlimbs from mice with mild, moderate, and high total joint Safranin‐O scores are shown. Scale bars = 100 μm. Sections were scored by a blinded observer for (b) the degree of Safranin‐O staining loss (high = OA, max score = 48) and (c) the size of osteophytes (high = OA, max score = 12). Analysis by Mann–Whitney test showed no significant difference between groups (*p* > .05) for either measure

Given that no effect of *p16* ^*INK4a*^ deletion was observed in aging‐associated OA, we sought to provoke a more severe and phenotypically homogeneous form of OA through DMM. Applying our genetic approach to a joint injury model was also motivated by the demonstration that *p16* ^*INK4a*^ expression increases with transection of the anterior cruciate ligament and that elimination of senescent cells can mitigate post‐traumatic OA (Jeon et al., 2017). DMM surgery was performed on littermate cohorts of *Acan* ^*tm1(cre/ERT2)Crm*^ *p16* ^*INK4a+/+*^ and *Acan* ^*tm1(cre/ERT2)Crm*^ *p16* ^*L/L*^ male mice at 12 months of age and we assessed cartilage degradation 8 weeks after surgery. Histological evidence of cartilage degradation was present in the medial compartment of DMM hindlimbs (Figure 5a). As expected, DMM surgery increased both the Safranin‐O and osteophyte scores as compared to the contra‐lateral control hindlimbs in both the *p16* ^*INK4a*^ intact and *p16* ^*INK4a*^ loss cohorts (*p* < .05, Figure 5b,c). As was the case for age‐induced OA, however, there was no difference in the degree of OA when comparing the DMM hindlimbs of *p16* ^*INK4a*^ loss mice to the DMM hindlimbs of *p16* ^*INK4a*^ intact mice (*p* > .05, Figure 5b,c). These results indicate that *p16* ^*INK4a*^ loss in chondrocytes is insufficient to protect from the development of age‐induced or post‐traumatic OA.

## 3 DISCUSSION

Advanced chronological age is the most prevalent risk factor for the development of OA. There is mounting evidence that the dysfunctional chondrocyte phenotype that emerges with aging and OA is characteristic of cellular senescence (McCulloch et al., 2017). While p16^INK4a^ is known to inhibit cellular proliferation with aging and senescence in many tissues, the lack of chondrocyte proliferation in vivo provides an opportunity to assess whether p16^INK4a^ plays a functional role in aging that is independent of cell cycle inhibition, as has been proposed in other physiological settings (Goel et al., 2017; Murakami et al., 2012). In this study, we found that *p16* ^*INK4a*^ expression is an effective biomarker of chondrocyte dysfunction despite not being an essential mediator of age‐related or injury‐induced OA. These results provide biological insight into the functional effects of increased *p16* ^*INK4a*^ expression with aging. Furthermore, our findings support translational work that seeks to identify and eliminate senescent cells from tissue compartments that drive age‐related disease.

The expression of *p16* ^*INK4a*^ increases with age in both murine and human cartilage. In murine cartilage, *p16* ^*INK4a*^ gene expression increased approximately 50‐fold from skeletal maturity at 4 months to either 18 or 22–27 months of age. In primary chondrocytes from human cadaveric donors without evidence of OA, *p16* ^*INK4a*^ and the related transcript *p14* ^*ARF*^ were the only genes tested that showed a significant correlation to age. Furthermore, we used Palbociclib treatment (mimicking high *p16* ^*INK4a*^ expression) to demonstrate that chondrocytes are responsive to CDK4/6 inhibition when cultured ex vivo under conditions that support proliferation. In line with reports of senescent features in other postmitotic cells such as neurons (Jurk et al., 2012) and osteocytes (Farr et al., 2016), our results demonstrate that the *INK4a/ARF* locus is potently activated with aging even in a cell type that is not affected by replicative exhaustion in vivo.

The possibility that increased *p16* ^*INK4a*^ expression in chondrocytes may contribute to age‐related OA independent of growth arrest led us to investigate the functional consequence of somatic inactivation in a murine model. We found that the somatic loss of *p16* ^*INK4a*^ in chondrocytes did not protect against either age‐related or post‐traumatic OA. This finding is interesting in light of the beneficial effect of selectively eliminating senescent cells from murine hindlimbs after anterior cruciate ligament transection (ACLT) (Jeon et al., 2017). ACLT is a more severe model than DMM (Fang & Beier, 2014), but the more likely explanation for the discrepancy is that our approach eliminated *p16* ^*INK4a*^ without affecting the SASP, whereas the senolytic compound reduced the production of SASP factors from the joint. Indeed, in some settings, p16^INK4a^ may even restrain the development of the SASP (Coppe et al., 2011). Together, these results support the interpretation that the effects of chondrocyte senescence on OA development are more likely to be driven by the SASP arm of senescence without the requirement of *p16* ^*INK4a*^ expression.

Age‐associated changes occur in many of the cells and tissues of the joint, which together play an active role in the development of OA through mechanisms such as the propagation of inflammatory cascades (Loeser, 2013). Of particular relevance is the recent finding that osteocytes exhibit features of cellular senescence with age, including expression of *p16* ^*INK4a*^ and SASP markers (Farr et al., 2016). Our in vivo approach focused on the role of *p16* ^*INK4a*^ in chondrocytes through the use of the *Acan* ^*tm1(cre/ERT2)Crm*^ allele that targets articular chondrocytes and meniscal cells (Henry et al., 2009), as well as a fraction of osteoblasts through direct conversion of hypertrophic growth plate chondrocytes (Ono, Ono, Nagasawa & Kronenberg, 2014; Zhou et al., 2014). Given our findings that interfering with *p16* ^*INK4a*^ expression did not alter SASP production, investigating *p16* ^*INK4a*^ loss in cell types with greater proliferative potential than chondrocytes may be of interest. For example, synovial fibroblasts and progenitor cells in the superficial zone of cartilage could be targeted for *p16* ^*INK4a*^ loss with the *Prg4* ^*tm1(GFP/cre/ERT2)Abl*^ allele (Kozhemyakina et al., 2015). Lineage tracing studies have suggested that subpopulations of synovial cells harbor regenerative potential (Decker et al., 2017) and a proliferative block in these cells through senescence may limit this capacity with aging. Our findings may have also been influenced by the development of medullary neoplasia with associated bone production in the *Acan* ^*tm1(cre/ERT2)Crm*^ *p16* ^*L/L*^ mice. This confounding factor may have limited the potential beneficial effects of *p16* ^*INK4a*^ loss in this cohort. While the articular cartilage was not affected, altered bone marrow function can cause indirect effects due to systemic differences such as extramedullary hematopoiesis.

Regardless of the functional role for p16^INK4a^ in OA development, investigating *p16* ^*INK4a*^ expression as a biomarker of chondrocyte dysfunction has important biological and translational implications due to the challenges of identifying tractable markers of senescence in vivo (Sharpless & Sherr, 2015). Despite the limitations of any particular marker of senescence, the emergence of chondrocytes demonstrating high *p16* ^*INK4a*^ expression, SASP, and SA β‐gal staining is indicative of the potential for senescence in this tissue compartment. Human chondrocytes with high expression of *p16* ^*INK4a*^ had reduced expression of Aggrecan and increased expression of the catabolic SASP factors *MMP‐1*,* MMP‐13*, and *IGFBP3*, even though expression of these genes had no relationship to age. Our interpretation is that high *p16* ^*INK4a*^ expression marks a subset of chondrocytes with the potential to cause tissue dysfunction through secretion of catabolic factors. The quantity of this subset increases during aging (thus the correlation of *p16* ^*INK4a*^ to age) and *p16* ^*INK4a*^ also serves as a biomarker that identifies chondrocytes with greater potential for secreting factors that drive tissue dysfunction (thus the correlation of *p16* ^*INK4a*^ to SASP markers). The utility of *p16* ^*INK4a*^ as a biomarker of molecular age in other cell types has been demonstrated by assessing the impact of lifestyle modifications such as smoking or exercise (Liu et al., 2009), predicting the risk for particular negative outcomes after chemotherapy (Demaria et al., 2017), and screening the quality of potential donor organs (Koppelstaetter et al., 2008). For chondrocytes, screening patients for low *p16* ^*INK4a*^ expression may improve the outcomes of autologous chondrocyte implantation procedures, as the success of this procedure requires avoidance of senescence to ensure sufficient expansion and subsequent re‐differentiation of the chondrocytes (Ashraf et al., 2016).

Further characterization of *p16* ^*INK4a*^ expression as a biomarker of chondrocyte senescence may also support the development of senolytic therapies for OA. The clinical development of senolytic therapies will require accurate identification of senescent cells before and after treatment (Kirkland et al., 2017). Identifying the patients most likely to respond to senolytics is particularly important for OA, as patient stratification for clinical trials is likely necessary to overcome the heterogenous nature and slow progression of the disease (Karsdal et al., 2016). As was demonstrated for age‐related osteoporosis, clearing senescent cells or directly inhibiting the SASP can change the balance of anabolic and catabolic processes in aged tissues (Farr et al., 2017). Senolytics are particularly attractive for OA because they have the potential to clear multiple cell types that secrete SASP factors into the joint space. Importantly, the periodic clearance of senescent cells from the joint was shown to have a long‐term benefit after ACLT despite a short half‐life of the compound (Jeon et al., 2017), indicating that sustained pharmacologic activity is not required. Intriguingly, this study also presented evidence that clearing *p16* ^*INK4a*^‐high cells may limit the development of age‐related spontaneous OA in addition to the more extensive studies in the context of post‐traumatic OA (Jeon et al., 2017). The ability to directly disrupt the age‐associated decline in tissue function that underlies most cases of OA would be a significant advance (Collins, Diekman & Loeser, 2018). Our findings on the role of *p16* ^*INK4a*^ expression in human and murine cartilage aging will help guide the development of therapies that seek to reduce the burden of senescence as a treatment for OA.

## 4 EXPERIMENTAL PROCEDURES

### 4.1 Generation of mouse colonies

All animal experiments were performed under protocols approved by the Institutional Animal Care and Use Committee of the University of North Carolina at Chapel Hill. The *Acan* ^*tm1(cre/ERT2)Crm*^ allele (Henry et al., 2009) (received from Dr. Benoit de Crombugghe, now available as stock #019148; Jackson Labs, Bar Harbor, ME, USA) was crossed to a *p16* ^*L*^ conditional allele (Monahan et al., 2010). For OA studies with *p16* ^*INK4a*^ intact (*Acan* ^*tm1(cre/ERT2)Crm*^ *p16* ^*INK4a+/+*^) and *p16* ^*INK4a*^ loss (*Acan* ^*tm1(cre/ERT2)Crm*^p16^L/L^) cohorts, male mice of C57BL/6 background were used due to the known development of hindlimb OA. For flow cytometry cell sorting, the loxP‐stop‐loxP ZsGreen reporter mouse (Gt(ROSA)26Sor^tm6(CAG‐ZsGreen1)Hze^/J stock # 007906; Jackson Labs) was crossed into mice with or without loss of p16^INK4a^. For lineage tracing and some flow cytometry cell sorting, mice containing *Acan* ^*tm1(cre/ERT2)Crm*^ were crossed to the loxP‐stop‐loxP tdTomato reporter mouse (Gt(ROSA)26Sor^tm14(CAG‐tdTomato)Hze^/J stock # 007914; Jackson Labs). For in vitro monolayer expansion studies, a constitutive type II collagen Cre driver (Col2a1‐Cre) was used to recombine the *p16* ^*L*^ conditional allele in chondrocytes without the necessity of tamoxifen induction (Sakai et al., 2001).

### 4.2 RNA isolation and qPCR from murine cartilage

Cartilage tissue was dissected from the proximal end of the femur from C57BL/6 mice with the following ages: 4, 10–12, 18, and 22–27 months. Tissue was placed in ceramic bead tubes (MP Bio, Santa Ana, CA, USA) containing Trizol (Thermo Fisher Scientific, Waltham, MA, USA) and isolated using a Precellys^®^ 24 homogenizer (Bertin Corp, Rockville, MD, USA). RNA was isolated using phenol chloroform extraction and NucleoSpin^®^ column clean‐up (Macherey‐Nagel, Düren, Germany). Reverse transcription was performed using the ImProm‐II™ system (Promega Corporation, Madison, WI, USA) according to the manufacturer's instructions and quantitative RT–PCR was performed with TaqMan™ Universal Master Mix on a QuantStudio™ 6 Flex machine (Applied Biosystems, Foster City, CA, USA). Custom TaqMan™ primers specific to murine *p16* ^*INK4a*^ (Assay ID: AIMSG0H; F: CGGTCGTACCCCGATTCAG; R: GCACCGTAGTTGAGCAGAAGAG; probe AACGTTGCCCATCATCA) and *p19* ^*ARF*^ (Assay ID: AIMSH0Y; F: TGAGGCTAGAGAGGATCTTGAGAAG; R: GTGAACGTTGCCCATCATCATC; probe: ACCTGGTCCAGGATTC) were used, with data normalized to murine Tbp as a housekeeping control (Mm00446973_m1; Applied Biosystems). For studies involving gene expression analysis of murine chondrocytes with and without p16 loss, chondrocytes were sorted based on Cre‐driven fluorescent reporters directly into Trizol, RNA was cleaned with NucleoSpin^®^ columns, and RNA reverse transcribed with qScript XLT reagent (Quantabio, Beverly, MA, USA). Taqman primer probes for *Mmp13* (Mm00439491_m1) and *Igfbp3* (Mm01187817_m1) were used for SASP analysis.

### 4.3 Culture of murine chondrocytes and senescence‐associated β‐galactosidase (SA β‐gal) analysis

For studies on cell expansion, chondrocytes from 3‐week‐old Col2a1‐Cre; loxP‐stop‐loxP ZsGreen mice were digested overnight using 0.4 mg/ml Collagenase P. Cells sorted as zsGreen‐positive chondrocytes were plated at 10,000 cells/cm^2^ and passaged weekly. In the final passage, some wells were stained for SA β‐gal (Cell Signaling Technologies, Danvers, MA) according to the manufacturer's recommendations. After DAPI counterstain, five matched bright field and fluorescent images were taken for each independent cell preparation to quantify the percentage of positively stained cells.

### 4.4 Tamoxifen injection and destabilization of the medial meniscus (DMM) surgery

At four months of age (and again at 12 months for the aging study), mice received three doses of 25 mg/kg tamoxifen (Sigma‐Aldrich, St. Louis, MO, USA) in corn oil (Sigma‐Aldrich) by intraperitoneal injection to activate the Cre recombinase. The DMM surgery (Glasson, Blanchet & Morris, 2007) was performed on mice at 12 months of age as described previously (Loeser et al., 2012). Briefly, mice were anesthetized with isoflurane, access to the joint space was obtained through a para‐patellar incision, and the medial meniscotibial ligament was transected using a scalpel. Mice recovered with normal movement and were sacrificed 8 weeks after the surgery.

### 4.5 Histological assessment of OA

Murine hindlimbs were dissected at sacrifice and fixed for 3–4 days in 4% paraformaldehyde (Electron Microscopy Sciences, Hatfield, PA, USA) at 4°C before decalcification in Immunocal for 3–4 days at room temperature (age‐related OA study) or 19% EDTA for 2 weeks at room temperature (DMM study). Joints were embedded in paraffin and coronal sections of 4–5 μm were taken from the mid‐coronal plane for histological assessment. Slides stained with Safranin‐O (proteoglycans)/Fast Green (collagen)/Hematoxylin (nuclei) were used to analyze a Safranin‐O score as previously described (McNulty et al., 2011). Briefly, the four quadrants of the load‐bearing region (medial and lateral sides of the femoral condyle and tibial plateau) were summed after grading on a 0–12 scale, with 12 representing complete loss of staining or tissue over more than 2/3 of the surface. An articular cartilage structure score was also generated as previously described (McNulty et al., 2011), which showed similar results to the Safranin‐O staining score but with less sensitivity to subtle changes in OA progression (data not shown). Slides stained with hematoxylin and eosin were used to assess osteophyte score, with each quadrant graded on a 0–3 scale for osteophyte size as previously described (Rowe et al., 2017). For immunohistochemistry with a primary antibody targeting tdTomato (cat # 600‐401‐379; Rockland Immunochemicals, Limerick, PA) or targeting Mmp‐13 (ab84594; Abcam, Cambridge, UK), antigen retrieval was performed with heated sodium citrate and sections were stained using the VECTASTAIN^®^ Elite ABC‐HRP kit (Vector Laboratories, Burlingame, CA).

### 4.6 Human chondrocyte isolation and qPCR

Human ankle tissue was provided by Dr. Susan Chubinskaya at Rush University Medical Center (Chicago, IL) through the Gift of Hope Organ and Tissue Donor Network (Elmhurst, IL). Donors with OA (Collins grade ≥ 3) were excluded. As previously described (Loeser, Pacione & Chubinskaya, 2003), cartilage tissue was dissected and sequentially digested with Pronase and Collagenase to obtain chondrocytes. Cell pellets were snapped frozen and stored at −80°C until RNA isolation with RNeasy columns (Qiagen, Hilden, Germany). RNA was reverse transcribed using the ImProm‐II™ system (Promega) and analyzed for gene expression as indicated in Table 1.

### 4.7 Cell cycle analysis of primary human chondrocytes

Isolated primary chondrocytes were plated in six‐well plates (Corning, Corning, NY, USA) at 20,000 cells/cm^2^ in DMEM/F12 media (Gibco, Life Technologies, Carlsbad, CA, USA) with 10% fetal bovine serum (Sigma‐Aldrich), penicillin/streptomycin (Gibco), and amphotericin B (Sigma‐Aldrich). For studies comparing young (24.25 ± 2.6 years old) and older donors (64 ± 2.1 years old), a 4‐hr pulse of EdU was added 22–26 hr after a media change to label cells in S phase. Cells were harvested by trypsinization, fixed in 1% paraformaldehyde, and permeabilized with 0.1% Saponin (Sigma‐Aldrich). Incubation was performed with Click‐It™ EdU Alexa Fluor™ 555 according to the manufacturer's instructions and DAPI was used to label DNA for assessment of 2n and 4n content. Cells were analyzed on either a CyAn flow cytometer (Beckman Coulter, Brea, CA) or Attune NxT (Thermo Fisher) flow cytometer.

### 4.8 CDK inhibition and siRNA treatment of primary chondrocytes

For assessment of cell cycle inhibition in five donors (67 ± 5.7 years old), 1 μm Palbociclib (PD‐0332991 HCl, ChemShuttle, Wuxi, China) or 50 nm Dinaciclib (Selleck Chem, Houston, TX) was delivered to chondrocytes for 26 hr, with the inclusion of EdU for the final 4 hr. For knockdown of the *INK/ARF* locus, chondrocytes from four donors underwent Amaxa Nucleofection (Lonza, Basel, Switzerland) for treatment with 1 μm siRNA SMARTPool (Dharmacon, Lafayette, CO) that targets both *p16* ^*INK4a*^ and *ARF* or a scrambled control. Gene expression for SASP markers was analyzed 3 days after transfection.

### 4.9 Statistical analysis

Statistical analysis and plotting were performed using Prism 7 (GraphPad, La Jolla, CA, USA) and flow cytometry data were processed with FCS Express (De Novo Software, Glendale, CA, USA). Data are plotted as individual points with bars indicating mean ± standard error of the mean (*SEM*). Correlations for human chondrocyte gene expression to age and p16^INK4a^ were performed using linear regression of data normalized to the housekeeping gene YWHAZ, with *r* ^2^ indicating goodness of fit. For Safranin‐O and osteophyte scores, data were analyzed using nonparametric tests (Kruskal–Wallis for one‐way ANOVA, Mann–Whitney test for comparing two unmatched groups, and Wilcoxon signed rank test for paired analysis of control vs. DMM limbs). All other analysis was performed on normally distributed data using *t* test or ANOVA with Tukey's post hoc analysis.

## CONFLICT OF INTERESTS

NES is a co‐founder of G1 therapeutics and HealthSpan Diagnostics. NES and RFL have been consultants for Unity Biotechnology. CSC is a consultant for Zoetis and Pfizer.

## AUTHORS’ CONTRIBUTIONS

BOD involved in research design, data collection, data analysis, and manuscript preparation. GAS analyzed and collected the data. JAC designed the research and collected the data. AKK collected the data and developed the system. SLS collected the data and developed the system. NKM analyzed and collected the data. CSC analyzed and collected the data. RFL involved in research design, data analysis, and manuscript preparation. NES involved in research design, data analysis, and manuscript preparation.
