# Supplementary material for: Expression of p16INK 4a is a biomarker of chondrocyte aging but does not cause osteoarthritis
Source: Aging Cell. 2018 May 9;17(4):e12771. doi: 10.1111/acel.12771 (PMC6052464; doi:10.1111/acel.12771)
Supplement: Supplementary file 1 [file ACEL-17-na-s001.pdf]

Supporting Information Figure 1

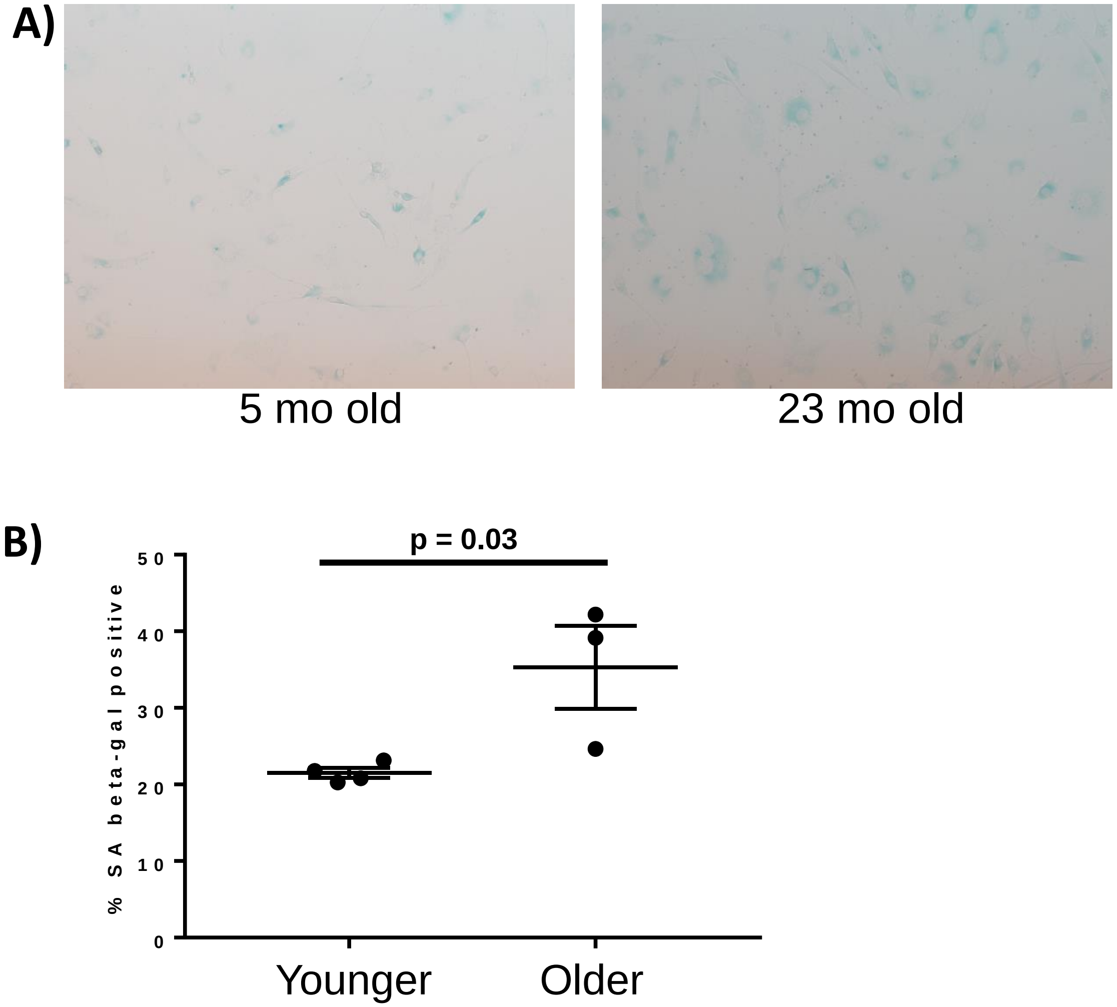

Figure S1. Effect of aging on senescence-associated β-galactosidase staining in murine chondrocytes. Chondrocytes from younger (5-9 months) or older (22-24 months) mice were sorted based on *Acan*$^{tm1(cre/ERT2)Crm}$-driven fluorescent reporter expression. After six days of monolayer culture, cells were fixed and stained for SA β-gal activity. (A) Representative images from 5-month-old and 23-month-old murine chondrocytes. (B) Quantification of the percentage of SA β-gal positive cells as counted by a blinded observer, with total cell counts achieved by DAPI counterstain. P-value by t-test shown.

Supporting Information Figure 2

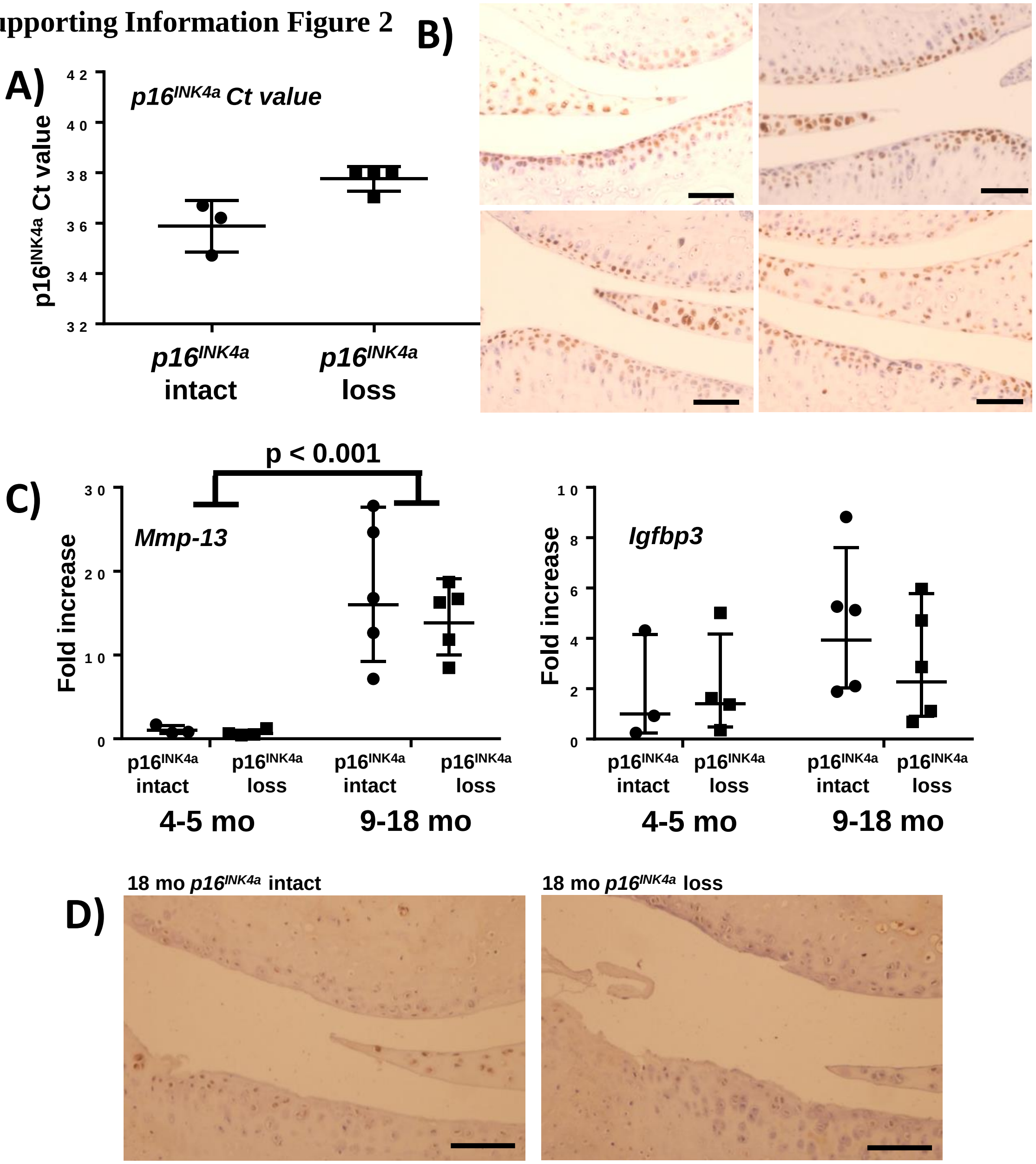

Figure S2. Effect of induced $p16^{INK4a}$ loss on the senescence-associated secretory phenotype. (A) Chondrocytes were analyzed one month after tamoxifen delivery by qPCR. The expression of $p16^{INK4a}$ (left) was undetectable for both replicates in three out of four $p16^{L/L}$ mice and set to a Ct value of 38 for plotting. (B) $Acan^{tm1(cre/ERT2)Crm}$-driven recombination of a lox-stop-lox tdTomato reporter allele was assessed with immunohistochemistry for tdTomato in four different mice. (C) Gene expression of *Mmp-13* (left) and *Igfbp3* (right) was analyzed in young and older mice with and without $p16^{INK4a}$ loss. (D) Protein production of Mmp-13 was evaluated with immunohistochemistry in 18 month-old mice with and without $p16^{INK4a}$ loss. Images shown are representative of three mice from each group.

Supporting Information Figure 3

A)

Neoplasia with bone deposition	$Acan^{tm1(cre/ERT2)Crm}$ $p16^{INK4a+/+}$ ($p16^{INK4a}$ intact)	$Acan^{tm1(cre/ERT2)Crm}$ $p16^{L/L}$ ($p16^{INK4a}$ loss)
DMM-induced OA (14 mo old)	0/15	11/13
Age-related OA (18 mo old)	2/20	9/12

B)

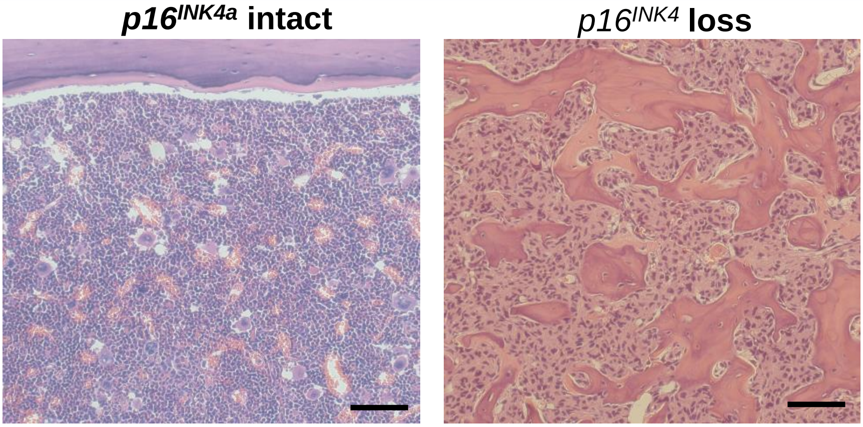

Figure S3: Intramedullary neoplasia accompanied by bone deposition. (A) The number of mice exhibiting neoplasia with intramedullary bone deposition by histologic assessment of the bone marrow of the tibia and femur. (B) Representative hematoxylin and eosin stained slides from the bone marrow of the femur in $p16^{INK4a}$ intact (normal) and $p16^{INK4a}$ loss (with neoplasia) mice at 18 months of age. Scale bars = 50 µm.

Supporting Information Figure 4

A)

B)

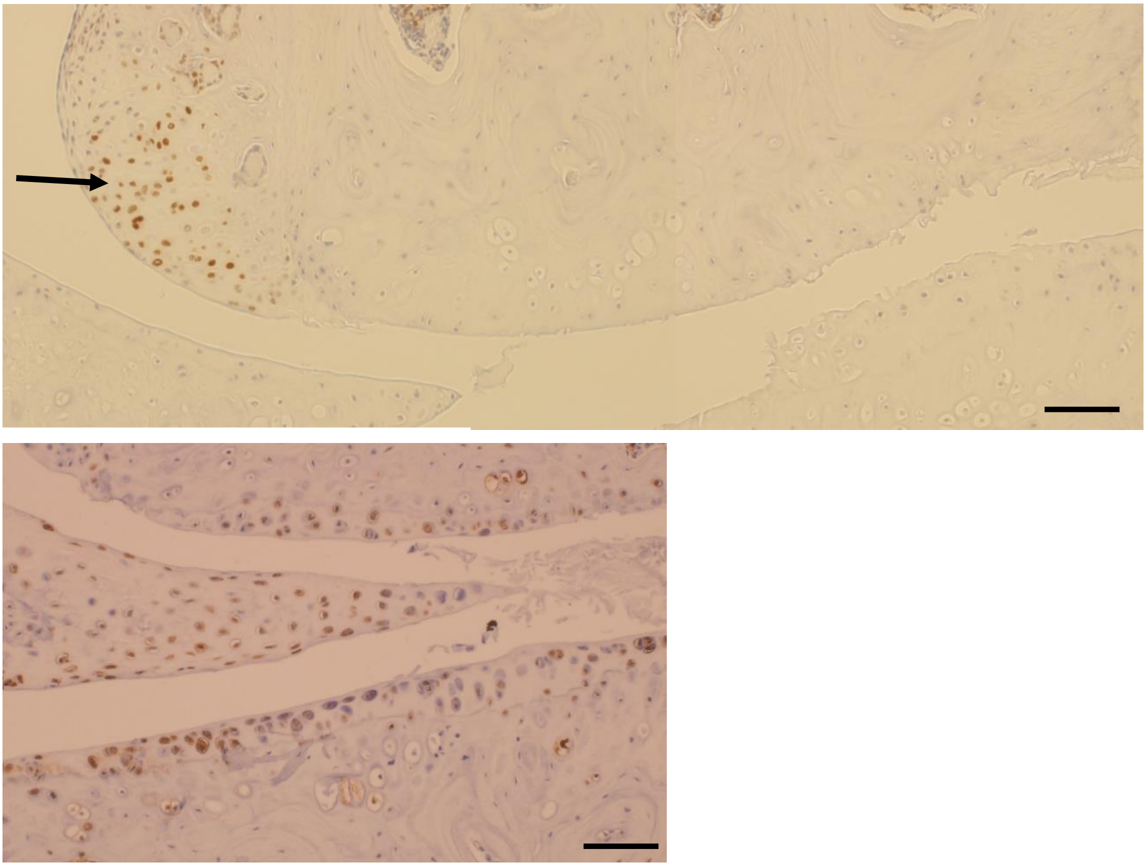

Figure S4: Long-term BrdU pulse and stability of *Acan*$^{tm1(cre/ERT2)Crm}$–driven reporter in cartilage. (A) 3 week BrdU pulse after destabilization of the medial meniscus (DMM) surgery with *p16*^{INK4a} loss. Representative image from results of 2 *p16*^{INK4a} intact and 3 *p16*^{INK4a} loss mice sacrificed 4 weeks after DMM surgery. Positive signal (brown stain) in area of early osteophyte formation is denoted by the arrow. Note that two images are overlayed to represent the joint surface. Scale bar = 50 µm. (B) Six-month follow up of recombination initiated in skeletally mice was assessed with immunohistochemistry to tdTomato in *Acan*$^{tm1(cre/ERT2)Crm}$;loxP-stop-loxP reporter mice. Representative image from results of 3 mice, scale bar = 50 µm.

Supporting Information Figure 5

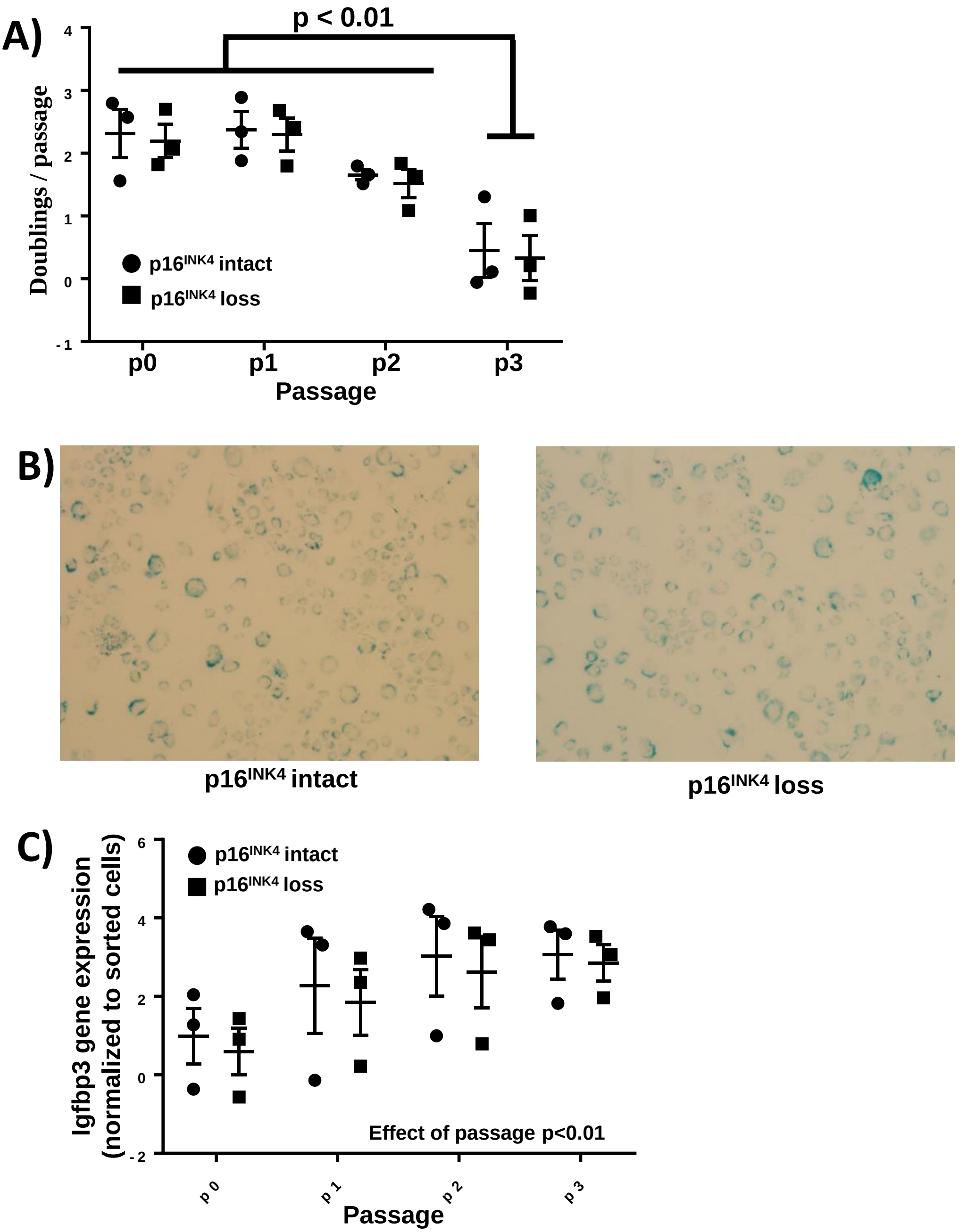

Figure S5. Effect of *$p16^{INK4a}$* loss on replicative senescence in expanded murine chondrocytes. (A) Expansion rates shown during extended passage of mice with and without Col2a1-Cre driven recombination of homozygous *$p16^{L}$* mice as compared to littermate controls. P values by ANOVA with Tukey's post-hoc shown. (B) Senescence-associated β-galactosidase staining of representative monolayer cultures during passage 3 with and without *$p16^{INK4a}$* loss. (C) *Igfbp3* gene expression during monolayer expansion with and without *$p16^{INK4a}$* loss. Two-way ANOVA showed significance for passage number but not *$p16^{INK4a}$* genotype.
